# Numerical Study on Oil Particle Enrichment in a Rectangular Microfluidic Channel Based on Acoustic Standing Waves

**DOI:** 10.3390/mi17010079

**Published:** 2026-01-07

**Authors:** Zhenzhen Liu, Jingrui Wang, Yong Cai, Yan Liu, Xiaolei Hu, Haoran Yan

**Affiliations:** 1 Anhui Province Engineering Laboratory of Intelligent Demolition Equipment, School of Mechanical Engineering, Anhui University of Technology, Ma’anshan 243032, China; zhenzhenliu@ahut.edu.cn (Z.L.); w19556633886@163.com (J.W.);; 2 Anhui Province Silicon-Based New Materials Characteristic Industry Innovation Research Institute, Chuzhou 233030, China; 3 Civil Aviation Key Laboratory of Aircraft Health Monitoring and Intelligent Maintenance, College of Civil Aviation, Nanjing University of Aeronautics and Astronautics, Nanjing 210016, China

**Keywords:** oil condition monitoring, particle enrichment, acoustic standing waves, microfluidic channel, acoustic radiation force

## Abstract

This study presents a method for enriching oil-suspended particles within a rectangular microfluidic channel using acoustic standing waves. A modified Helmholtz equation is solved to establish the acoustic field model, and the equilibrium between acoustic radiation forces and viscous drag is described by combining Gor’kov potential theory with the Stokes drag model. Based on this force balance, the particle motion equation is derived, enabling the determination of the critical particle size necessary for efficient enrichment in oil-filled microchannels. A two-dimensional standing-wave microchannel model is subsequently developed, and the influences of acoustic, fluidic, and particle parameters on particle migration and aggregation are systematically investigated through theoretical analysis and numerical simulations. The results indicate that when the channel dimension and acoustic wavelength satisfy the half-wavelength resonance condition, a stable standing-wave field forms, effectively focusing suspended particles at the acoustic pressure nodes. Enrichment efficiency is found to be strongly dependent on inlet flow velocity, particle diameter, acoustic frequency, temperature, and particle density. Lower flow velocities and larger particle sizes result in higher enrichment efficiencies, with the most uniform and stable pressure distribution achieved when the acoustic frequency matches the resonant channel width. Increases in temperature and particle density enhance the acoustic radiation force, thereby accelerating the aggregation of particles. These findings offer theoretical foundations and practical insights for acoustically assisted online monitoring of wear particles in lubricating oils, contributing to advanced condition assessment and fault diagnosis in mechanical systems.

## 1. Introduction

During the operation of complex mechanical systems, prolonged contact and relative motion between lubricated surfaces inevitably generate a significant amount of wear debris. These particles are transported by the lubricating oil [1,2] and carry direct information regarding component degradation. Specifically, changes in their size, morphology, and concentration reflect active wear mechanisms and the overall operating condition of the equipment [3,4,5]. Consequently, oil analysis based on wear particle characterization has become a key technique for condition monitoring and early fault diagnosis [6,7], with broad applications in aerospace systems [8], wind turbines [9,10], and hydraulic systems [11]. However, the stochastic motion of wear particles in oil during operational monitoring of the systems significantly degrades the performance of conventional oil debris sensors, such as the optical sensors [12,13,14], inductive sensors [15,16] and electrostatic sensors [17,18]. This limitation becomes particularly critical during the incipient wear stage, where a large number of fine particles (typically below 50 μm) are randomly dispersed in the lubricating oil [19]. Therefore, the efficient online enrichment of these particles is a critical prerequisite for accurately and reliably monitoring equipment health and wear conditions.

To meet the need for efficient enrichment of suspended particles, acoustic manipulation within microfluidic systems has attracted growing attention in recent years. This technique combines high integration, low sample consumption, and precise controllability, providing a promising strategy for microscale particle enrichment [20,21]. In such systems, external physical fields, including electric [22,23,24], magnetic [25], optical [26,27,28,29], and acoustic fields [30], have been employed to induce controlled particle migration and aggregation. Among these approaches, techniques leveraging acoustic streaming and standing acoustic waves are particularly attractive due to their non-contact nature, minimal mechanical damage, and capacity for continuous on-chip operation [31,32]. Standing acoustic waves generate a spatially periodic pressure field in the microchannel, imposing acoustic radiation forces that drive particles toward pressure nodes or antinodes. This force field induces directed migration and clustering, enabling the selective enrichment of particles [33]. Acoustic manipulation has been widely explored in biomedicine and environmental monitoring [34,35], for example, in cell concentration [36], microplastic detection [37], and blood component separation [38].

Building on these principles, several studies have investigated particle aggregation in acoustic fields using both experimental and numerical methods. Settnes and Bruus [39] developed a theoretical framework for acoustic radiation forces and acoustic streaming acting on small particles in viscous fluids. Muller et al. [40] analyzed the competition between acoustic radiation forces and acoustic streaming, showing how their relative importance depends on particle size. Soliman et al. [41] realized continuous separation of different blood cell types in standing-wave microchannels. Khan et al. [42] proposed a surface acoustic wave-based particle sorting model, clarifying how acoustic frequency and pressure amplitude affect the aggregation efficiency of particles with different shapes. Lu et al. introduced a filter-free acoustofluidic manipulation chip, which provides a pretreatment solution for wear debris analysis and lubricant condition monitoring [43].

Despite significant progress, most acoustofluidic studies have focused on biological cells or polymer microspheres dispersed in low-viscosity aqueous solutions. Metallic wear particles in lubricating oils differ markedly, exhibiting much higher densities, stronger inertial effects, and rougher surfaces, all of which substantially change their responses to acoustic radiation forces and hydrodynamic drag. In addition, the higher viscosity and lower density of lubricating oils lead to pronounced coupling between acoustic propagation and viscous dissipation, altering both the acoustic field distribution and the resulting radiation force. It should be noted that particle enrichment in acoustic fields is governed by the combined effects of acoustic parameters (frequency and amplitude), fluid properties (flow velocity, viscosity, and density), and particle characteristics (size and density). However, their coupled effects on particle migration dynamics and enrichment efficiency remain poorly understood [44,45].

In response to these challenges, this study systematically investigates the online enrichment of oil-suspended wear particles using standing acoustic waves. Unlike previous studies that focus on isolated parameters or aqueous suspensions, our approach accounts for the coupled effects of acoustic excitation, flow conditions, particle properties, and oil temperature on enrichment efficiency. The key contributions of this study are: (i) development of a coupled acoustofluidic-particle dynamics model to describe the migration and enrichment of metallic wear particles in viscous lubricating oils; (ii) identification of the critical particle size for effective enrichment under realistic flow and acoustic conditions; (iii) systematic evaluation of the influence of key operational parameters—such as flow velocity, acoustic frequency, particle density, and oil temperature—on enrichment efficiency.

In response to the aforementioned issues, this study systematically investigates an online enrichment method for oil particles based on acoustic standing waves. The structure of this paper is arranged as follows: Section 2 presents the theoretical foundations and mathematical modeling. Section 3 provides a detailed description of the numerical simulation procedure. Section 4 presents the results and discussion, which include the distribution of acoustic flow fields and the effect of five factors on the particle enrichment. The conclusions are shown in Section 5. These results provide a theoretical foundation for online acoustic enrichment of wear debris, thereby facilitating the development of intelligent fault detection and health monitoring strategies for lubricated machinery.

## 2. Theoretical Foundations and Mathematical Modeling

### 2.1. Governing Equations of the Hydrodynamic and Acoustic Field Frequency-Domain Models

In this study, the acoustic field is described using a frequency-domain pressure acoustic model. The governing equation is formulated in terms of a modified Helmholtz equation, which can be expressed as:

(1) $$\nabla \cdot \left(-\frac{1}{\rho_{c}}\nabla p_{t}\right)-\frac{k_{eq}^{2}}{\rho_{c}}p_{t}=Q_{m},$$

where $p_{t}$ denotes the total acoustic pressure, consisting of the static and perturbation pressures; $p_{t}=p+p_{b}$, $\rho_{c}$ is the effective density; $Q_{m}$ represents the volumetric source term; and the effective wavenumber $k_{eq}^{2}$ is defined as:

(2) $$k_{eq}^{2}={\left(\frac{\omega }{c_{c}}\right)}^{2}-k_{z}^{2},$$

where ω is the angular frequency, $k_{z}$ is the wavenumber in the z-direction, and $c_{c}$ is the corrected speed of sound. To account for fluid viscosity and thermal conduction effects, the corrected sound speed $c_{c}$ and the effective density $\rho_{c}$ are defined as follows:

(3) $$c_{c}=c{\left(1+i\omega \frac{\delta }{c^{2}}\right)}^{\frac{1}{2}}$$

(4) $$\rho_{c}=\frac{\rho c^{2}}{c_{c}^{2}},$$

where ρ and c are the medium density and the ideal speed of sound, respectively. The parameter δ, which is associated with viscous and thermal losses, is given by:

(5) $$\delta =\frac{1}{\rho }\left(\frac{4}{3}\mu +\mu_{B}+\frac{\left(\gamma -1\right)k}{c_{p}}\right),$$

where μ is the shear viscosity, $\mu_{B}$ is the bulk viscosity, γ is the ratio of specific heats, k is the thermal conductivity, and $c_{p}$ is the specific heat capacity at constant pressure. The governing equations, which comprehensively model the energy dissipation of acoustic waves in viscous fluids, are formulated to determine the standing-wave field distribution inside the microchannel.

In this study, the liquid flow within the microchannel is described using an incompressible laminar flow model. The governing equations for fluid dynamics are the steady-state, incompressible Navier–Stokes equations, which consist primarily of the conservation of momentum and mass. Their mathematical formulations are as follows:

(6) $$\rho \left(u\cdot \nabla \right)u=\nabla \cdot \left[-pI+K\right]+F$$

(7) ∇⋅u=0

Here, ρ denotes the fluid density, u is the velocity field, p represents the pressure, and F is the body force term. The viscous stress tensor K is expressed based on the Newtonian fluid assumption as follows:

(8) $$K=\mu \left(\nabla u+(\nabla u{)}^{T}\right)$$

Here, μ is the dynamic viscosity coefficient. The above equations are used to calculate the flow velocity and pressure distribution in the microchannel, serving as the base flow field in the acoustic-fluid-particle multiphysics coupling.

### 2.2. Theory of Acoustic Radiation Force and Acoustic Streaming Drag

**Figure 1:**
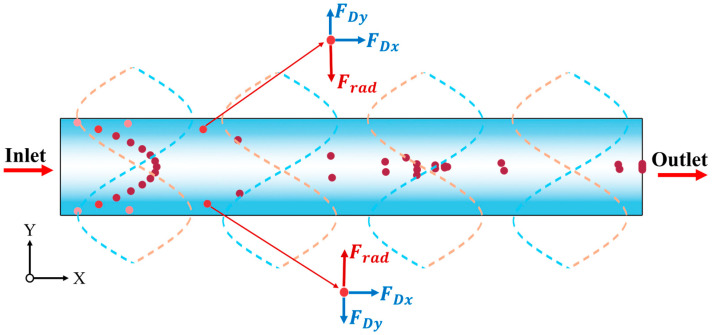
Schematic diagram of the conditions within the microchannel (forces acting on particles, fluid flow direction) and acoustic field characteristics (the central white region represents the pressure node, while the blue regions on both sides correspond to antinodes; the particles with higher velocity are dark, and the particles with lower velocity are light).

The motion of a particle in an acoustic standing-wave field is primarily governed by the combined effects of the acoustic radiation force and the viscous drag (Stokes drag) exerted by the fluid [46]. The forces acting on particles in the standing wave field are shown in Figure 1.

(a) Acoustic Radiation Force Model

For particles whose radius is much smaller than the acoustic wavelength (i.e., satisfying the Rayleigh scattering condition a≪λ), the radiation force can be described using the Gor’kov potential theory. According to Gor’kov’s formulation, the time-averaged acoustic radiation force acting on a single spherical particle can be expressed as:

(9) $$F_{rad}=-\nabla U,$$

where U is the Gor’kov potential function; based on its analytical derivation, the acoustic radiation force acting on the particle can be written as:

(10) $$F_{rad}=-2\pi r_{p}^{3}\left[\frac{1}{3}\kappa_{s}Re\left(f_{0}^{s1}p^{*}\nabla p\right)-\frac{1}{2}\rho Re\left(f_{1}^{s1}u^{*}\cdot \nabla u\right)\right],$$

where $r_{p}$ is the particle radius; ρ and $k_{s}$ denote the fluid density and bulk modulus, respectively; p and u represent the first-order acoustic pressure and velocity fields. The dimensionless scattering coefficients $f_{0}^{sl}$ and $f_{1}^{sl}$ characterize the particle’s response to compressibility and density contrasts, and are defined as follows:

(11) $$f_{0}^{sl}=1-\kappa_{s}$$

(12) $$f_{1}^{sl}=\frac{2\left(\tilde{\rho }-1\right)}{2\tilde{\rho }+1}$$

Here, $\tilde{\rho }=\rho_{p}/\rho$ represents the density ratio between the particle and the fluid, and $\kappa_{s}=k_{p}/\kappa_{s}$ is the compressibility ratio, with $\kappa_{s}=1/(\rho c^{2})$ and $\kappa_{p}=1/(\rho_{p}c_{p}^{2})$, where c and $c_{p}$ are the sound speeds in the fluid and particle, respectively. The direction of the acoustic radiation force is determined by the particle’s acoustic contrast factor Φ:

(13) $$\Phi =\frac{1}{3}f_{0}^{s1}+\frac{1}{2}f_{1}^{s1}$$

Particles move toward the pressure nodes when Φ>0, whereas they migrate toward the pressure antinodes when Φ<0.

(b) Stokes Drag Model

When particles move through a fluid in a microchannel, they experience viscous resistance, primarily in the form of Stokes drag. This force is proportional to the relative velocity between the particle and the fluid, with the Stokes drag $F_{D}$ defined as follows:

(14) $$F_{D}=\frac{1}{\tau_{p}}m_{p}\left(u-v\right)$$

Here, v is the instantaneous particle velocity, u is the fluid velocity, $m_{p}$ is the particle mass, and $\tau_{p}$ is the particle relaxation time, representing the timescale over which the particle velocity responds to changes in the fluid flow. The particle relaxation time $\tau_{p}$ is defined as:

(15) $$\tau_{p}=\frac{\rho_{p}d_{p}^{2}}{18\mu }$$

Here, $\rho_{p}$ is the particle density, $d_{p}$ is the particle diameter, and μ is the dynamic viscosity of the fluid. Consequently, particles with larger density or size exhibit an increased relaxation time $\tau_{p}$, reducing their responsiveness to fluid flow. Conversely, smaller particles more readily follow the fluid motion within the flow field.

### 2.3. Particle Motion Equation and Critical Particle Size

Under the coupled acoustic–flow field, the migration dynamics of a particle are governed jointly by the acoustic radiation force $F_{\mathrm{rad}}$ and the Stokes drag $F_{D}$. The governing dynamical equation can be expressed as:

(16) $$m_{p}\frac{dv}{dt}=F_{rad}+F_{D}$$

Considering the scalar components along the driving direction and introducing a characteristic velocity U to represent the fluid-induced hindrance, a particle will migrate toward and accumulate at the pressure node once the acoustic radiation force exceeds the fluid drag. The steady-state force balance can therefore be expressed as:

(17) $$F_{rad}\approx F_{D}$$

Under the one-dimensional approximation, the amplitude of the acoustic radiation force is commonly expressed as:

(18) $$F_{rad}∼4\pi a^{3}\phi E_{ac}k,$$

where a is the particle radius, ϕ is the acoustic contrast factor, $E_{\mathrm{ac}}$ denotes the time-averaged acoustic energy density, and k is the wave number. At low Reynolds numbers, the Stokes drag can be approximated as:

(19) $$F_{D}\approx 6\pi \eta aU$$

Here, η is the dynamic viscosity of the fluid, and U is the characteristic flow velocity. By equating the two expressions and solving for the particle radius a, the critical value $a_{c}$ is obtained as follows:

(20) $$a_{c}=\sqrt{\frac{3\eta U}{2\phi E_{ac}k}}$$

When the particle radius $a>a_{c}$, the acoustic radiation force dominates over the viscous drag, enabling the particle to migrate toward the pressure node under the action of the radiation force. When $a<a_{c}$, the viscous drag becomes dominant, causing the particle to follow the fluid flow and making effective acoustic enrichment difficult to achieve.

## 3. Numerical Simulations

### 3.1. Model Construction and Parameter Settings

Since the computational representation of the three-dimensional rectangular channel is consistent with its two-dimensional counterpart, we established a two-dimensional model to reduce computational cost. To generate a stable, high-energy standing acoustic wave in this channel, the acoustic wavelength must match the channel dimension along the propagation direction so that the half-wavelength resonance condition is satisfied. This requirement couples the acoustic excitation frequency to the channel width W, which must satisfy W=λ/2, where λ is the acoustic wavelength. Using the relation c=fλ between the sound speed c and the wavelength λ, the corresponding resonance frequency f is obtained as:

(21) $$f=\frac{c}{2W}$$

When an excitation frequency of f=5 MHz and a sound speed of approximately c=1500 m/s in the fluid, the corresponding channel width is W=150 μm. Accordingly, the 2D microchannel model employed in this work has dimensions L×W=900 μm×150 μm, which is the baseline model.

To clarify the relationships between the physical phenomena and control parameters in the model, we define a set of baseline values and corresponding ranges. For the acoustic frequency, we cover the range that can excite half-wavelength resonance and related acoustic effects. Several microchannel sizes are specified, each with its own resonant frequency, to investigate how sound waves at different frequencies interact with the fluid and particles. For the flow field, we prescribe several inlet velocities to study how the flow rate affects particle transport. Particle diameter is a key factor governing the forces and motion of particles, so multiple size groups are selected to evaluate the role of size differences throughout the entire process. We also vary particle material properties to analyze how changes in density affect the response to the acoustic radiation-force gradient and the resulting trajectories. The temperature ranges from 10 to 50 °C. With the temperature dependence of fluid properties taken into account, we investigate how temperature jointly influences the acoustic-field distribution, hydrodynamic behavior and particle-focusing efficiency.

Accurate specification of material properties in the model is essential, because these parameters directly determine the fidelity of the simulated acoustic propagation, fluid flow and particle motion. In this study, the acoustic excitation frequency was selected to satisfy the half-wavelength resonance condition characteristic of typical microchannel dimensions, while the excitation amplitude was constrained within ranges readily achievable by piezoelectric transducers in microfluidic applications. The selected ranges of flow velocity and temperature are practically feasible. Despite these simplifications, the chosen parameter settings ensure that the simulated acoustic fields, flow behaviors, and particle dynamics remain physically meaningful and relevant to practical application scenarios. The basic model parameters are listed in Table 1.

### 3.2. Mesh Generation and Independence Verification

The accuracy of acoustic finite-element simulations strongly depends on mesh resolution. To resolve both the standing-wave field and the viscous boundary layer, a hybrid meshing strategy was adopted, combining unstructured bulk meshes with refined boundary-layer meshes. Within the channel interior, free triangular elements were employed to accommodate the complex distributions of the flow and acoustic fields, while layered boundary elements were generated along the microchannel walls. These refined boundary-layer elements enable accurate resolution of the near-wall velocity profile and particle motion. Two meshing criteria were imposed. First, the acoustic field was resolved with at least 6–10 elements per wavelength to ensure accurate representation of wave propagation. Second, the number of boundary-layer layers and their thickness distribution were selected to capture the steep gradients within the viscous sublayer with sufficient fidelity. The mesh size was determined from the acoustic wavelength to balance accuracy and computational cost. With a sound speed of c=1500 m/s and a driving frequency of $f_{0}=5\;MHz$, the acoustic wavelength is $\lambda =c/f_{0}=300\;\mu m$. Accordingly, the maximum element size in the bulk region was limited to λ/6=50 μm. Additionally, eight progressively refined boundary-layer layers were applied along the upper and lower channel walls to resolve the sharp velocity and acoustic pressure gradients in the near-wall region.

To assess the sensitivity of the numerical solution to mesh resolution, the acoustic field in the channel was computed using four meshes with maximum element sizes of 50 μm, 30 μm, 10 μm and 5 μm. Mesh convergence was evaluated using the average acoustic energy density *E* in the channel region, defined as the sum of the time-averaged kinetic and potential acoustic energy densities:

(22) $$E=\bar{E_{k\iota n}}+\bar{E_{pot}}=\frac{1}{4}\rho_{0}|v_{0}{|}^{2}+\frac{1}{4}\frac{|p_{0}{|}^{2}}{\rho_{0}c^{2}}$$

Here, $\bar{E_{k\iota n}}$ denotes the time-averaged kinetic energy density, representing the kinetic energy stored due to the oscillation of fluid particles; $\bar{E_{pot}}$ denotes the time-averaged potential energy density, representing the energy stored due to compression and rarefaction of the medium. $\rho_{0}$ is the static density of the medium, c is the speed of sound in the medium, $v_{0}$ is the complex amplitude of particle velocity, and $p_{0}$ is the complex amplitude of the acoustic pressure.

**Figure 2:**
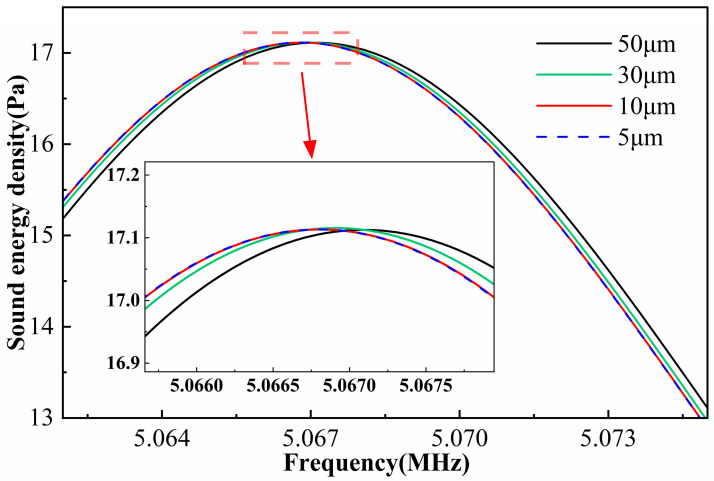
Average acoustic energy density of the model under different mesh sizes.

Figure 2 shows the variation in acoustic energy density in the vicinity of the resonant frequency for different mesh resolutions. As the mesh is refined, the energy-density curves converge toward a single profile. The 50 μm mesh exhibits pronounced deviations over the entire frequency range, indicating insufficient resolution. For the 30 μm mesh, the discrepancy relative to the finer meshes (10 μm and 5 μm) is significantly reduced, although small differences are in the amplitude. When the maximum element size is reduced to 10 μm, the results almost coincide with those obtained using the 5 μm mesh, demonstrating that mesh independence is essentially achieved and the numerical solution has converged. Accordingly, a maximum element size of 10 μm is adopted in this study to strike a balance between accuracy and computational cost.

### 3.3. Determination of the Resonant Frequency

**Figure 3:**
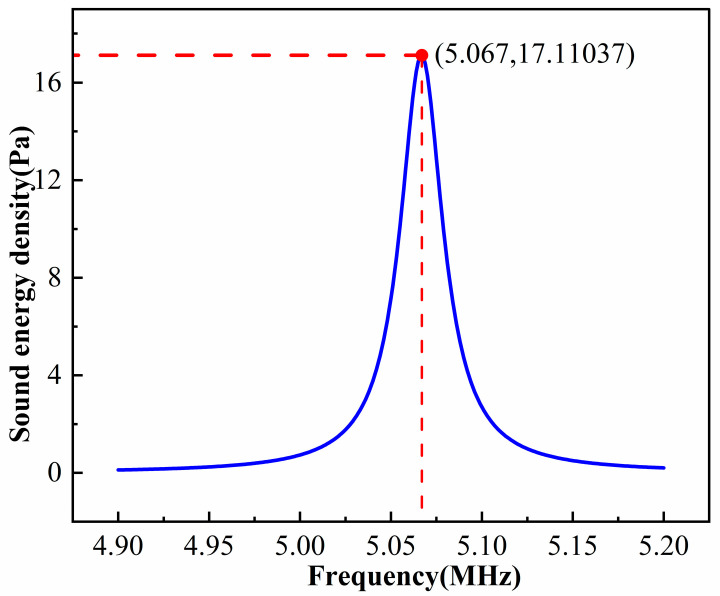
Average acoustic energy density of the model at different excitation frequencies.

In theory, when the channel width and acoustic frequency satisfy the half-wavelength resonance condition, a standing wave with a specific vibration mode is excited in the channel. At this frequency, the system exhibits its strongest response to external excitation and the highest energy-transfer efficiency. This frequency is defined as the resonance frequency. However, the resonance frequency obtained numerically often deviates from this theoretical value. To determine the resonance frequency accurately, we first specify the channel width W and the sound speed c in the fluid. Using Equation (21), we calculate the theoretical half-wavelength resonance frequency f. We then perform a frequency sweep around this value and compute the average acoustic energy density at each frequency. As an example, for the baseline channel with W=150 μm, we sweep a range of 300 kHz around f=5 MHz with a step of 1 kHz. After solving, we use post-processing to obtain the average acoustic energy density in the fluid domain at each frequency. The resulting curve is shown in Figure 3. A pronounced peak appears at about 5.067 MHz, which is taken as the resonance frequency of the system.

### 3.4. Model Validation

**Figure 4:**
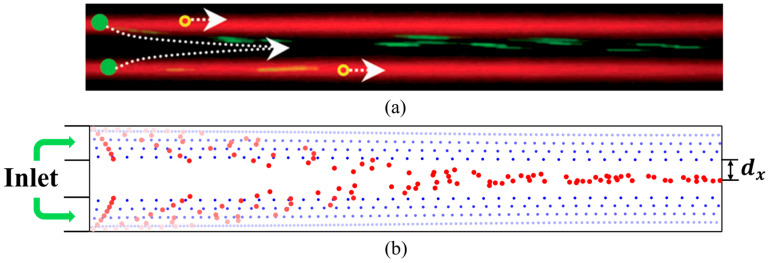
Comparison between experimental observations and numerical simulations of particle motion along the flow direction: (**a**) Experimental results of Shi et al.; (**b**) simulation results (Red represents the larger particles, and blue represents the smaller particles).

To validate the numerical model, we simulated particles of two diameters injected from both sides of the channel (Figure 4b). We then compared the simulated trajectories with the experiments of Shi et al. [47] under the same initial and boundary conditions. In their experiments, large particles (4.17 μm) migrated from the side streams toward the central pressure node of the standing wave. In comparison, small particles (0.87 μm) experienced much weaker acoustic radiation forces and were mainly carried by the flow to the side outlets. The simulations reproduced this behavior. Driven by the acoustic radiation force in the longitudinal direction, the large particles gradually migrate toward the centerline of the channel as the fluid flow transported them. Consequently, they become concentrated at the center of the channel by the outlet. Small particles stayed mainly near the channel sides, consistent with the observed separation. We further made a quantitative comparison using the separation time and separation distance. The separation time was defined as the interval from particle entry to the formation of a stable, dense band of large particles at the center. The separation distance $d_{x}$ was defined as the gap between the large-particle and small-particle bands. In the experiments, large particles completed migration in about 360 ms, and the separation distance was 26 μm. The model predicted a separation time of about 350 ms and a separation distance of 27 μm. This close agreement supports the accuracy of the numerical model.

These comparisons demonstrate that the numerical model reproduces particle separation in a standing acoustic wave accurately, confirming its ability to capture acoustically driven particle dynamics. Nevertheless, it should be noted that the present 2D model does not account for certain three-dimensional effects, such as end-wall boundary influences, axial acoustic attenuation, and fully three-dimensional acoustic streaming vortices. These effects may introduce quantitative deviations in particle trajectories and enrichment efficiency in practical three-dimensional oil-monitoring systems. Despite these limitations, the 2D model provides a computationally efficient and physically insightful framework for systematically investigating the coupled influences of flow velocity, excitation frequency, particle size, temperature, and material properties. The results are therefore expected to capture the dominant trends and mechanisms governing acoustic enrichment, while serving as a useful reference for future three-dimensional simulations and experimental validation.

## 4. Results and Discussion

### 4.1. Distribution of Acoustic and Flow Fields in the Model

**Figure 5:**
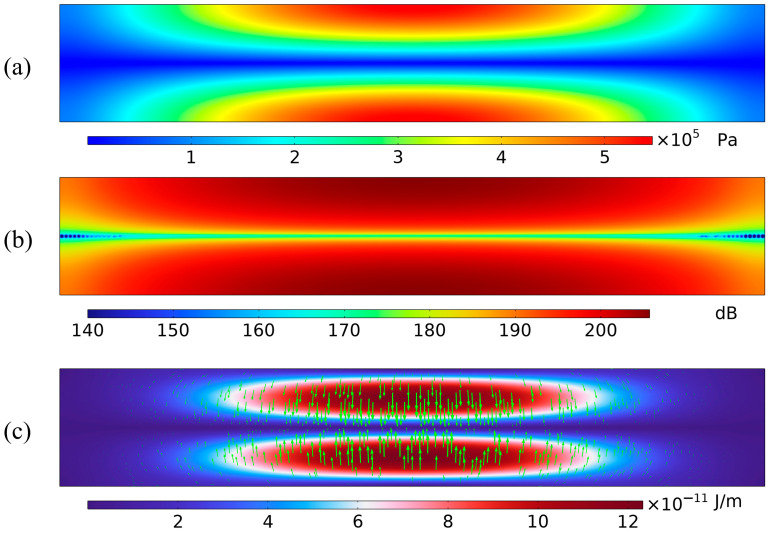
Acoustic field characteristics within the microchannel: (**a**) Acoustic pressure contour at the resonant frequency; (**b**) Sound pressure level contour at the resonant frequency; (**c**) Acoustic radiation force distribution on 5 μm particles at the resonant frequency (the green arrow represents the acoustic radiation force).

In the two-dimensional microchannel standing-wave model used in this study, the channel width and acoustic frequency satisfy the half-wavelength resonance condition. Under this condition, a stable standing wave is formed in the channel. The acoustic pressure exhibits antinodes at the upper and lower walls, and a node at the center of the channel. Because the standing-wave field has a spatial pressure gradient, the acoustic radiation force on the particles points toward the pressure node. Using the baseline model with L×W=900 μm×150 μm as an example, Figure 5 shows the acoustic field at resonance. Figure 5a shows the sound-pressure distribution in the microchannel. Pressure antinodes appear at the upper and lower walls, and a pressure node is located at the center of the channel. This pattern indicates a half-wavelength standing wave in the channel. The sound pressure varies periodically across the width: the amplitude is high near the walls and approaches zero at the center. Figure 5b shows the sound-pressure-level (SPL) distribution, which confirms these features. The SPL is highest near the walls and lowest at the center, forming a clear and symmetric lateral pressure gradient. This gradient reflects the variation in acoustic energy density across the channel and is the source of the acoustic radiation force. Figure 5c shows the distribution of the acoustic radiation force on the particles. The force vectors point from the walls toward the center, that is, from high-pressure regions to the low-pressure region. Thus, the net force in the standing-wave field drives particles toward the pressure node. As the pressure gradient increases, the radiation force becomes stronger, and the force vectors concentrate near the node, indicating stable particle focusing. When particles are suspended in this standing-wave field, they move along the pressure gradient toward the channel center and finally gather near the pressure node at the outlet, where they form a stable band and become effectively enriched.

**Figure 6:**
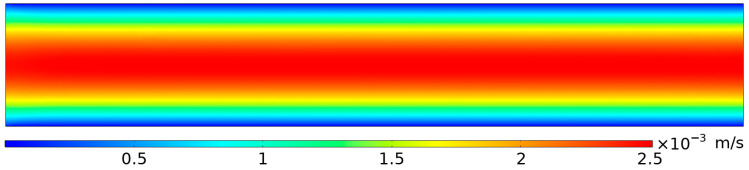
Velocity field distribution within the microchannel.

The fluid flow field provides the background motion that carries particles in the microchannel. Its accuracy directly affects the calculation of key forces on the particles, such as viscous drag and acoustic radiation force. Figure 6 shows the steady-state velocity field. It has a typical laminar profile, where the velocity is highest at the channel center and decreases to zero at the walls, resulting in an approximately parabolic distribution.

To quantify differences in particle enrichment performance, an explicit criterion for “completion of enrichment” is defined: enrichment is considered complete when all particles have entered the central region spanning one-third of the channel width. The x-coordinate at which the particle band first reaches this central one-third is defined as the enrichment completion position, and its distance from the outlet is denoted by D. The enrichment efficiency η is then used as a performance index and is calculated as follows:

(23) $$\eta =\frac{D}{L}$$

A larger value of η indicates that particles require a shorter distance within the microchannel to achieve effective focusing, thus reflecting a higher particle enrichment efficiency.

### 4.2. Effect of Oil Flow Velocity

Under an applied acoustic field, particle aggregation depends not only on the standing-wave pattern but also on the inlet flow velocity. To examine how flow rate affects particle focusing, we couple a laminar-flow module to the standing-wave acoustic model in a multiphysics framework. Several inlet velocities are specified to simulate particle trajectories and final distributions under different flow conditions.

**Figure 7:**
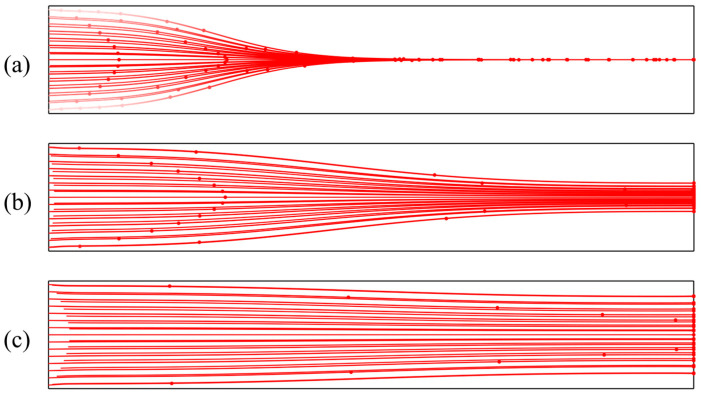
Particle trajectories under different inlet flow velocities: (**a**) 0.001 m/s; (**b**) 0.005 m/s; (**c**) 0.02 m/s.

At low inlet flow velocities, the hydrodynamic drag force exerted on particles is relatively weak. Consequently, particles have sufficient time to be dominated by the acoustic radiation force, allowing them to migrate rapidly toward the acoustic pressure node and form a stable aggregation band. Under these conditions, the aggregation position is distinct with a narrow bandwidth, indicating high stability in particle distribution, as shown in Figure 7a. However, as the inlet flow velocity increases, both fluid shear and drag forces intensify, while the residence time of particles within the acoustic field decreases. Due to insufficient migration time, some particles fail to reach the pressure node before exiting the channel. This results in reduced aggregation efficiency and a broadening of the aggregation band, as illustrated in Figure 7b. At higher flow velocities, convective effects dominate. The acoustic radiation force cannot effectively overcome the hydrodynamic drag, causing particles to be flushed out of the channel with the main flow without forming a discernible aggregation band, as depicted in Figure 7c.

### 4.3. Effect of Particle Diameter

**Figure 8:**
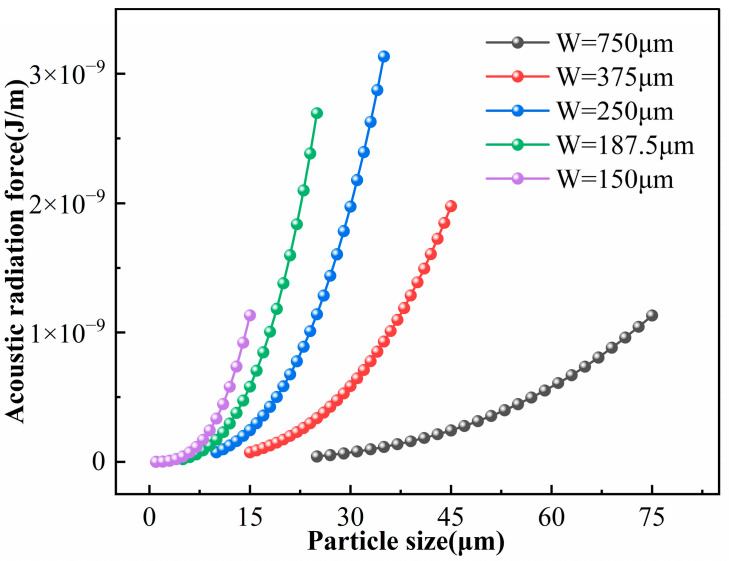
Acoustic radiation force on particles with different diameters in microchannels of different sizes.

Particle diameter is a key parameter controlling enrichment efficiency in the microchannel. According to Equation (20), when the particle radius $a>a_{c}$, particle motion is dominated by the acoustic radiation force and particles migrate toward the pressure nodes or antinodes; when $a<a_{c}$, acoustic streaming primarily governs particle motion. The present study focuses on aggregation behavior in the radiation-force-dominated regime ( $a>a_{c}$). According to Equation (10), the acoustic radiation force $F_{\mathrm{rad}}$ is proportional to the particle volume $\left(F_{\mathrm{rad}}\propto a^{3}\right)$; therefore, the radiation force increases nonlinearly with particle size. Figure 8 shows the relationship between the average acoustic radiation force and particle diameter at the resonant frequencies corresponding to different channel widths. All curves display a pronounced nonlinear increase, confirming that the acoustic radiation force is strongly enhanced with increasing particle size.

**Figure 9:**
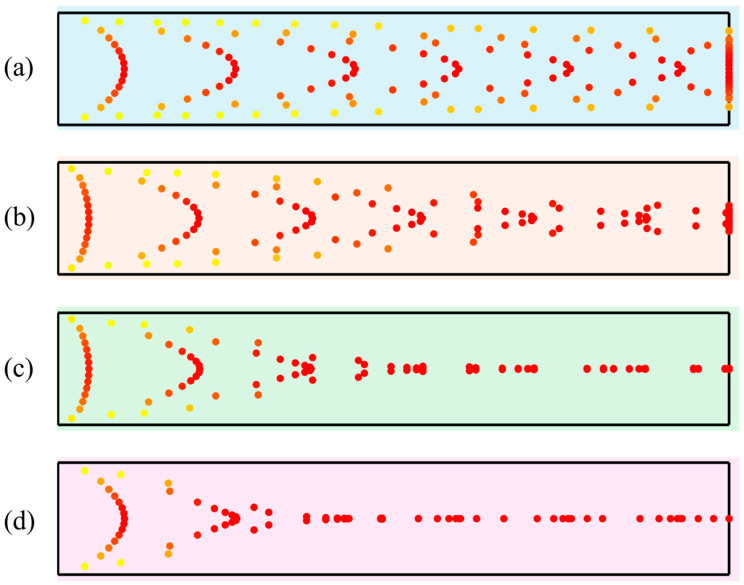
Particle trajectories for different diameters (red: particles with higher velocity; orange: particles with medium velocity; yellow: particles with lower velocity): (**a**) 1 μm; (**b**) 2 μm; (**c**) 4 μm; (**d**) 6 μm.

**Figure 10:**
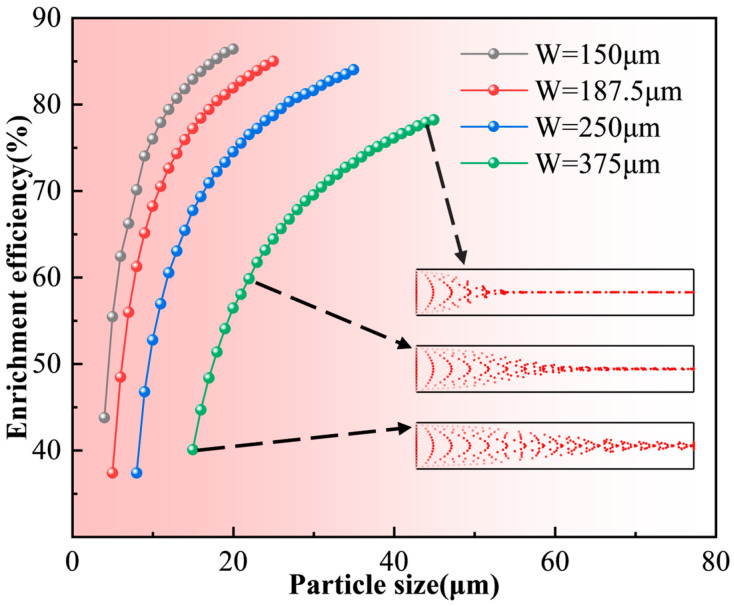
Enrichment efficiency of particles with different diameters in microchannels of varying sizes.

For the basic model with dimensions L×W=900 μm×150 μm, Equation (21) indicates a critical particle diameter of approximately 1 μm. Using the control variable method, particle trajectories for different diameters were calculated by varying only the particle size, as shown in Figure 9. As shown in Figure 9a, particles with a diameter of 1μm exhibit no distinct aggregation characteristics under the acoustic field due to their proximity to the critical size threshold. Consequently, they fail to form a stable enrichment zone within the channel. This indicates that the acoustic radiation force acting on small particles is too weak to overcome hydrodynamic drag for effective aggregation. As the particle diameter increases (2 μm, 4 μm, and 6 μm, corresponding to Figure 9b–d), the coupling between the particles and the acoustic field is significantly enhanced. Once the particle size exceeds the critical value, the acoustic radiation force increases with diameter, causing the particles to aggregate in specific regions rapidly. Regarding trajectory morphology, 2 μm particles show a preliminary aggregation trend, although the enrichment zone remains broad. For 4 μm particles, the aggregation paths are more concentrated, showing significantly improved trajectory convergence. In contrast, 6 μm particles form a dense enrichment band near the channel inlet with nearly overlapping trajectories. This demonstrates that larger particles are dominated by strong acoustic radiation forces, allowing them to overcome fluid disturbances and achieve rapid, precise enrichment efficiently. Figure 10 illustrates the enrichment efficiency for particles of different diameters at the resonance frequencies of various channel sizes. It reveals a positive correlation between particle size and efficiency: larger particles achieve earlier and tighter aggregation, resulting in significantly higher enrichment efficiency.

From the above results, it can be concluded that under half-wavelength resonance conditions and with all other parameters held constant, larger particles experience significantly stronger acoustic radiation forces than smaller particles within the same microchannel (Since particle migration is primarily driven by the acoustic radiation force, the subsequent analysis focuses on particle enrichment efficiency from the perspective of this force). Consequently, larger particles exhibit higher migration velocities and reach a steady-state distribution more rapidly. The particle enrichment efficiency increases with increasing particle diameter. Conversely, when the particle size is below the critical threshold, hydrodynamic drag dominates. This causes particles to follow the fluid flow, making it difficult to achieve effective capture.

### 4.4. Effect of Excitation Frequency

**Figure 11:**
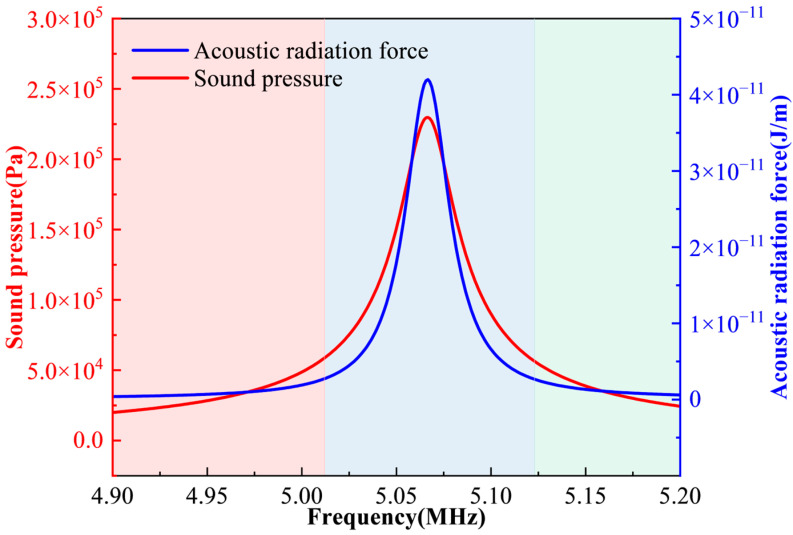
Acoustic pressure and radiation force in the model near the resonance frequency.

**Figure 12:**
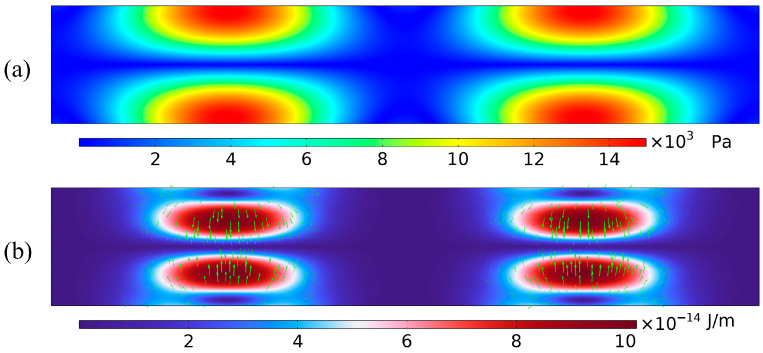
Distribution of acoustic field characteristics in the microchannel under non-half-wave resonance conditions: (**a**) Acoustic pressure distribution under non-half-wave resonance conditions; (**b**) Acoustic radiation force distribution in the channel at 6 MHz (the green arrow represents the acoustic radiation force).

To investigate the effect of frequency variation on particle enrichment, the resonance matching between the acoustic frequency and the channel width must be taken into account. In practical systems, changes in frequency disrupt this resonance condition, causing significant variations in the acoustic pressure distribution, which in turn affect the distribution of acoustic radiation forces and particle aggregation behavior. To systematically study this effect, two simulation strategies were designed in this work:

First, for the basic model with dimensions L×W=900 μm×150 μm, the excitation frequency was varied while keeping the channel width W constant to observe changes in the acoustic field structure and particle migration behavior within the microchannel. As shown in Figure 11, a clear standing wave structure with stable pressure nodes and antinodes forms only when the frequency satisfies the half-wave resonance condition with respect to the channel width—specifically, when the frequency is near the resonant frequency (blue region in Figure 11). In this regime, the acoustic pressure and the acoustic radiation force exerted on particles are significant, allowing particle motion to be dominated by the acoustic radiation force. When the frequency deviates from the resonance range (red and green regions in Figure 11), the standing wave structure remains intact; however, the weakened acoustic pressure and radiation force result in reduced aggregation efficiency or a complete failure to aggregate. As the frequency increases further, the half-wave resonance condition is no longer met. Consequently, the standing wave structure collapses, and the acoustic radiation force becomes weak and directionally unstable, preventing particle aggregation. Figure 12 illustrates the acoustic field structure in the microchannel and the acoustic radiation force exerted on the particles at 6 MHz.

**Figure 13:**
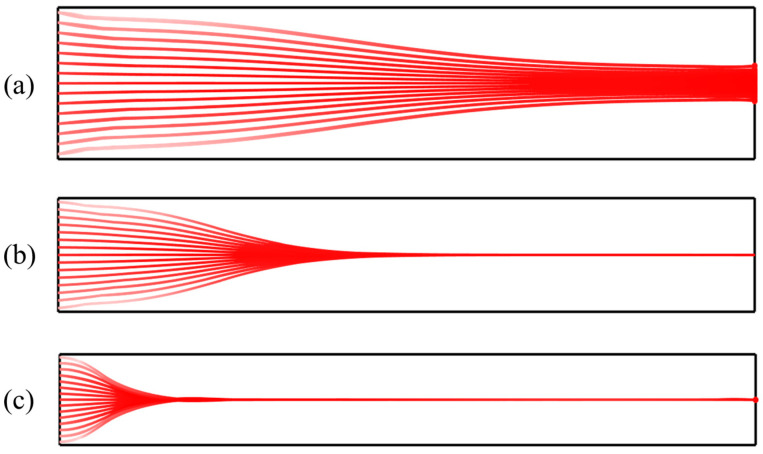
Particle trajectories in microchannels of different widths: (**a**) W=250 μm; (**b**) W=187.5 μm; (**c**) W=150 μm.

Secondly, while keeping other conditions constant, the channel width was adjusted simultaneously with the frequency to maintain the half-wave resonance condition. This allowed for an analysis of the acoustic radiation force and particle migration behavior under resonant states. Under these conditions, a stable standing wave distribution is consistently formed within the acoustic field, despite variations in excitation frequency. Simulation results in Figure 13 show that smaller channels correspond to higher resonance frequencies, leading to increased acoustic pressure and radiation forces. Additionally, the standing wave wavelength decreases, shortening the distance between pressure nodes and reducing the particle migration path. These factors collectively accelerate the enrichment rate, resulting in a more compact aggregation band. This trend is corroborated by Figure 8 and Figure 10, which show that under half-wave resonance conditions, particles of the same size experience greater acoustic radiation forces and exhibit higher enrichment efficiency in smaller channels. This indicates that channels with higher resonance frequencies and smaller widths possess stronger acoustic field energy, thereby reducing the critical particle diameter.

High-frequency, small-dimension microchannels are effective for manipulating smaller particles. In contrast, low-frequency (and larger) channels produce weaker acoustic fields, which provide insufficient radiation force for small particles but still allow for manipulation of larger ones. In summary, high-frequency, small microchannels are ideal for enriching smaller particles, while low-frequency, larger channels are more suitable for larger particles.

### 4.5. Effect of Oil Temperature

**Figure 14:**
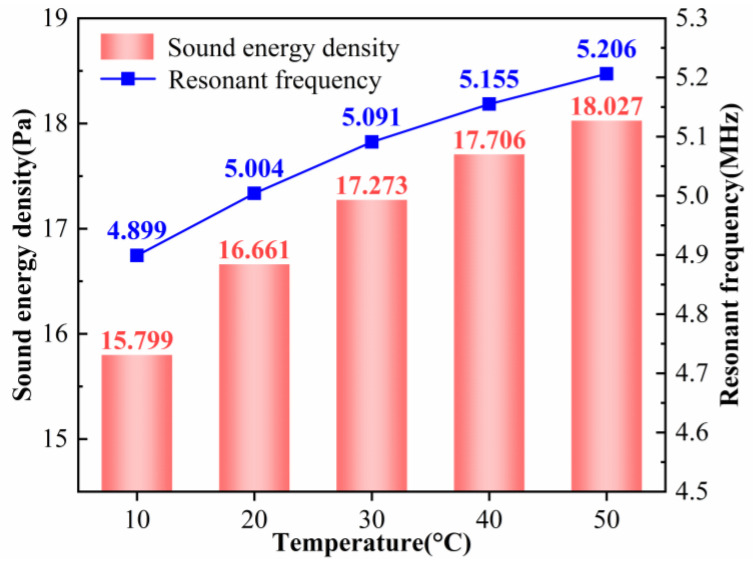
Resonant frequency and sound energy density of the model at different temperatures.

**Figure 15:**
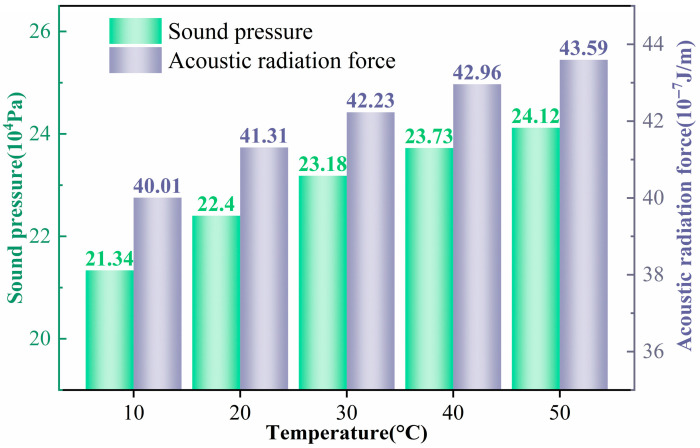
Sound pressure within the channel and the acoustic radiation force acting on the particle at different temperatures.

According to the acoustic radiation force expression in Equation (10), temperature does not appear explicitly, but it can influence the flow properties and thus modify the forces acting on the particles. Combining the simulation results in Figure 14 and Figure 15, it is found that when the temperature increases from 10 °C to 50 °C, the resonance frequency rises from approximately 4.899 MHz to 5.206 MHz (about 6.3%). At the same time, the average acoustic energy density increases from 15.799 Pa to 18.027 Pa (14.1%). The acoustic pressure amplitude in the channel increases from $21.34\times 10^{4}$ Pa to $24.12\times 10^{4}$ Pa (13.0%), and the acoustic radiation force on the particles increases from $40.01\times 10^{-7}$ J/m to $43.59\times 10^{-7}$ J/m (8.95%). These results indicate that higher temperatures enhance the acoustic energy and pressure amplitude in the resonant cavity, thereby increasing the magnitude of the acoustic radiation force exerted on the particles. Physically, temperature changes the thermophysical properties of the fluid (sound speed, density and viscosity), leading to two main effects: (i) slight modifications of acoustic coupling and resonance conditions, which shift the resonance frequency upward; and (ii) changes in energy transmission efficiency and damping, which increase the acoustic energy density and pressure amplitude under the same input conditions. In addition, as the temperature rises, the fluid’s viscosity generally decreases, thereby reducing the viscous resistance. The simultaneous increase in radiation force and decrease in drag facilitate particle migration and enrichment.

The influence of temperature on particle aggregation can be summarized in three aspects. (1) Higher aggregation efficiency and speed: As the acoustic radiation force increases and viscous resistance decreases, particles migrate toward the pressure node more rapidly, shortening the time required to reach a steady focused state. (2) Stronger enrichment: Higher acoustic pressure and energy density maintain a stronger converging effect at the node, leading to a higher local particle concentration in the final aggregation region. (3) Broader controllable particle-size range: For a given acoustic drive, the enhanced radiation force improves the ability to overcome thermal fluctuations and viscous drag for smaller particles, thereby reducing the critical particle size and extending the lower limit of manipulable particle size. It should be noted that temperature changes may require simultaneous adjustment of the excitation frequency to preserve optimal resonance. Moreover, at elevated temperatures, the possible onset of thermal convection and strong nonlinear coupling effects must be considered, as they may distort the standing-wave pattern and compromise the stability of particle enrichment.

### 4.6. Effect of Particle Density

To examine how particle material properties affect aggregation in a standing acoustic wave field, five representative particle materials were selected for comparison: polystyrene ($\rho_{p}=1050\;kg/m^{3}$, $c_{p}=2400\;m/s$), aluminum ($\rho_{p}=2700\;kg/m^{3}$, $c_{p}=6420\;m/s$), zirconia ($\rho_{p}=5680\;kg/m^{3}$, $c_{p}=6800\;m/s$), iron ($\rho_{p}=7870\;kg/m^{3}$, $c_{p}=5950\;m/s$), and copper ($\rho_{p}=8960\;kg/m^{3}$, $c_{p}=4700\;m/s$). All simulations were carried out under identical conditions, with the acoustic resonance frequency set to 5.067 MHz and the channel width maintained at 150 μm. As indicated by the theoretical analysis, the acoustic radiation force on the particles depends strongly on material parameters such as density and sound speed, and its direction is governed by the acoustic contrast factor Φ in Equation (13). When Φ>0, particles migrate toward the pressure nodes, whereas for Φ<0, they move toward the pressure antinodes.

**Figure 16:**
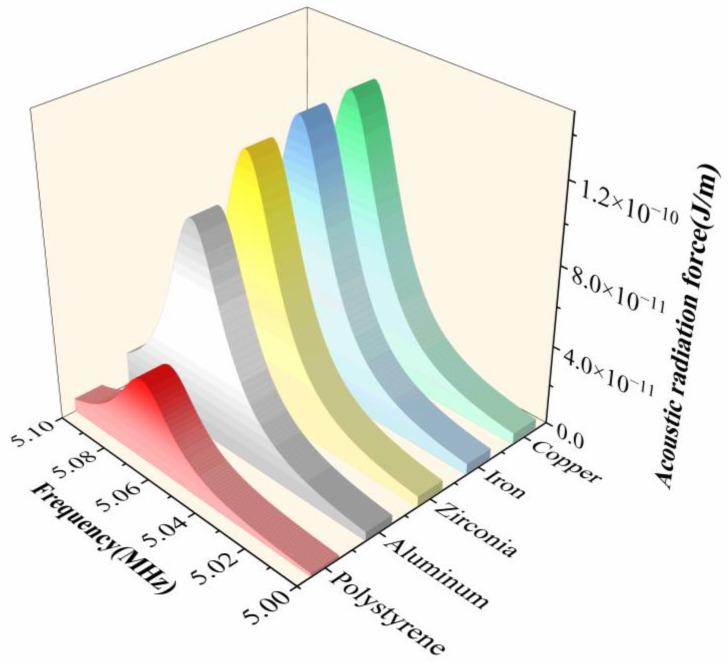
Acoustic radiation force on particles with different properties.

The acoustic contrast factor varies significantly among different particle materials, resulting in distinct differences in both the position and rate of aggregation. From Equations (12) and (13), the contrast factor Φ is directly related to the density ratio $\rho_{p}/\rho$. When the particle density is only slightly higher than that of the fluid, $f_{1}^{sl}$ is small and Φ is positive but weak, so the radiation force is relatively low. As $\rho_{p}$ increases, $f_{1}^{sl}$ and thus Φ rise, strengthening the radiation force. When $\rho_{p}$ becomes much larger than ρ, $f_{1}^{sl}$ approaches 1 and Φ tends toward an upper limit, so the radiation force no longer increases indefinitely but approaches saturation. Figure 16 shows the acoustic radiation force for particles with different material properties. For polystyrene particles, the density is only slightly higher than the fluid density, although the sound speed in the solid is much higher than that in the fluid. With Φ>0, these particles are driven toward the pressure nodes and form a relatively broad accumulation region around the nodes. Aluminum, zirconia, iron, and copper particles have much higher densities and sound speeds, yielding larger Φ values and hence stronger radiation forces. They migrate more rapidly toward the nodes and form narrower, more concentrated bands.

Overall, increasing particle density strengthens the influence of the acoustic radiation force, leading to faster aggregation and more compact accumulation. However, when particle density significantly exceeds that of the fluid, the radiation force reaches a saturation point, and the system stabilizes, with no further significant improvement in aggregation performance.

## 5. Conclusions

This work presents an acoustic streaming-based method for the online enrichment of oil-suspended particles. A two-dimensional standing wave model, incorporating Gor’kov potential and Stokes drag, reliably captures size-dependent particle migration and aggregation in microchannels. The study systematically demonstrates that enrichment is strongly influenced by particle diameter, inlet flow rate, acoustic frequency, temperature, and particle density, with larger and denser particles focusing more efficiently near the channel center under half-wavelength resonance. Flow-induced drag competes with acoustic forces, highlighting the need to balance enrichment efficiency and throughput. Temperature and fluid properties further modulate acoustic forces, affecting the critical particle size and aggregation dynamics. Overall, achieving efficient and stable particle enrichment requires a coordinated optimization of channel geometry, excitation frequency, flow conditions, and particle characteristics. Although this study focuses on oil-filled microchannels, the findings are also relevant to other fluid systems exhibiting similar acoustic and hydrodynamic characteristics. Future work will involve experimental validation to further confirm the numerical predictions, as well as investigations into three-dimensional channel geometries, nonlinear acoustic effects, and more complex fluid systems, with the aim of extending the applicability of acoustofluidic enrichment techniques.

## Figures and Tables

**Table 1:** Fundamental parameters of the model.

Parameter	Unit	Value
Temperature	K	300.31
Flow velocity	m/s	0.001
Bulk viscosity of fluid	Pa·s	$2.49\times 10^{-3}$
Dynamic viscosity of fluid	Pa·s	$8.92\times 10^{-4}$
Density of fluid	$kg/m^{3}$	996.45
Sound velocity in fluid	m/s	1500
Pressure wave velocity of particles	m/s	2400
Shear wave velocity of particles	m/s	1150

## Data Availability

The data that support the findings of this study are available from the corresponding author upon reasonable request.
